# Neurodevelopmental Disorders Associated with Gut Microbiome Dysbiosis in Children

**DOI:** 10.3390/children11070796

**Published:** 2024-06-28

**Authors:** Alejandro Borrego-Ruiz, Juan J. Borrego

**Affiliations:** 1 Departamento de Psicología Social y de las Organizaciones, Universidad Nacional de Educación a Distancia (UNED), 28040 Madrid, Spain; a.borrego@psi.uned.es; 2 Departamento de Microbiología, Universidad de Málaga, 29071 Málaga, Spain; 3 Instituto de Investigación Biomédica de Málaga y Plataforma en Nanomedicina-IBIMA, Plataforma BIONAND, 29010 Málaga, Spain

**Keywords:** children, gut microbiome, neurodevelopmental disorders, ASD, ADHD, Tourette syndrome, cerebral palsy, fetal alcohol spectrum disorders, genetic NDDs

## Abstract

The formation of the human gut microbiome initiates in utero, and its maturation is established during the first 2–3 years of life. Numerous factors alter the composition of the gut microbiome and its functions, including mode of delivery, early onset of breastfeeding, exposure to antibiotics and chemicals, and maternal stress, among others. The gut microbiome–brain axis refers to the interconnection of biological networks that allow bidirectional communication between the gut microbiome and the brain, involving the nervous, endocrine, and immune systems. Evidence suggests that the gut microbiome and its metabolic byproducts are actively implicated in the regulation of the early brain development. Any disturbance during this stage may adversely affect brain functions, resulting in a variety of neurodevelopmental disorders (NDDs). In the present study, we reviewed recent evidence regarding the impact of the gut microbiome on early brain development, alongside its correlation with significant NDDs, such as autism spectrum disorder, attention-deficit/hyperactivity disorder, Tourette syndrome, cerebral palsy, fetal alcohol spectrum disorders, and genetic NDDs (Rett, Down, Angelman, and Turner syndromes). Understanding changes in the gut microbiome in NDDs may provide new chances for their treatment in the future.

## 1. Introduction

The human body hosts over 10^12^ microbial cells, peaking in abundance within the enteric compartment, thereby constituting a sophisticated ecosystem referred to as the gut microbiome (GM) [1]. In the present work, the terms microbiota and microbiome are used interchangeably and synonymously; however, the microbiome is a broad concept that encompasses the full spectrum of microorganisms, the collection of genomes of all the microorganisms, and the microbial structural elements, metabolites, and environmental conditions [2]. The GM develops after birth, and it continues developing throughout the life of the host, from infancy to old age [3,4]. Members of the GM can be indigenous or transient microorganisms belonging to the domains Archaea, Bacteria, and Eukarya, but they also include viruses [5]. The composition of the microbiome reaches a state of homeostasis among all its members, forming complex trophic interrelations between them and their human host [6]. Several factors can cause shifts in this delicate microbial balance, consequently perturbing the homeostatic condition of the GM and provoking a state of dysbiosis [7].

The GM–brain axis refers to the interconnection of biological systems that permits a reciprocal communication between the gut bacteria and the brain, involving the nervous, endocrine, and immune systems [8,9]. Prenatal stress or prenatal maternal stress during pregnancy affects fetal brain development and changes in GM composition [10,11]. Several clinical and preclinical studies suggest that GM dysbiosis in the perinatal period may be involved in this process through the GM–brain axis, provoking several mental and behavioral outcomes in children, including attention-deficit/hyperactivity disorder (ADHD), autism spectrum disorder (ASD), schizophrenia, neuropsychiatric disorders, emotional dysregulation, anxiety disorders, aggressivity, and depressive states [5,12,13,14,15,16]. In addition, this axis seems to possess a pivotal role in the modulation of various brain processes, including myelination [17,18], microglial maturation [19], and neuronal plasticity [20,21,22,23] and eventually influences complex behaviors [21,24,25]. On the other hand, prenatal and postnatal stress have been associated with hypothalamic–pituitary–adrenal (HPA) axis dysregulation, resulting in increased cortisol levels and shifts in both the immune system and the GM [26,27,28].

The aim of this narrative review was to investigate the existing literature regarding how signals from the GM may impact neuroplasticity and brain development in childhood. In addition, neurodevelopmental disorders (NDDs) in children such as ASD, ADHD, Tourette syndrome, cerebral palsy, fetal alcohol spectrum disorders, and genetic NDDs (Rett, Down, Angelman, and Turner syndromes) were reviewed in order to know their GM composition and function changes.

## 2. Fetal Gut Microbiome

Early childhood is a key step in the establishment of the GM, with mode of birth, initial life feeding, and antibiotic exposure being the most important factors influencing the GM shaping [29]. The estimated time required to form a mature and functional GM is approximately 3 years [30,31]. However, the structure and composition of the GM are continuously and dynamically changing throughout life, depending on physiological and infectious diseases, drug consumption, dietary habits, or lifestyle, to name a few factors [32,33].

Until the beginning of the 21st century, it was believed that the neonatal gut was a sterile ecosystem (the “sterile womb paradigm”), starting its microbial colonization at birth. In recent years, this concept has changed due to the finding of bacterial cells or bacterial DNA in the umbilical cord blood, placenta, and meconium from the cesarean section (C-section) of healthy newborns [34,35,36]. Some authors have proposed the hypothesis of in utero colonization [37,38]; indeed, prenatal probiotics ingested by pregnant mothers have been detected in the placenta and in the stool of term newborns [39,40].

After birth, new microbial communities colonize the neonatal gut depending on several factors, such as birth mode, feeding of newborn, and use of antibiotics during infancy [31,41,42]. Other factors such as diet, maternal metabolic status and age, hereditary aspects, and lifestyle have also been suggested [43]. Another significant factor is the gestation period, given that the microbiome of preterm infants may contain potentially pathogenic microorganisms, be less diverse, and be depleted in short-chain fatty acids (SCFAs) [44,45,46]. In preterm neonates, the predominant bacteria found belonged to the phyla Pseudomonadota and Bacillota [46], to the families *Enterobacteriaceae* and *Enterococcaceae* [44], and to the genera *Lactobacillus*, *Clostridium*, *Escherichia*, *Klebsiella*, *Streptococcus*, *Staphylococcus*, and *Veillonella* [47,48].

The GM of vaginally delivered infants closely resembles the mother’s vaginal microbiota [49]. Conversely, infants born by C-section acquire microbial taxa similar to those present on human skin, alongside opportunistic pathogens often associated with healthcare environments [42,50]. The predominant bacterial composition of the GM in vaginally delivered neonates are *Bifidobacterium* and *Collinsella* (phylum Actinomycetota); *Clostridium*, *Lactobacillus*, *Streptococcus*, and *Veillonella* (phylum Bacillota); *Bacteroides*, *Parabacteroides*, and *Prevotella* (phylum Bacteroidota); *Sneathia* (phylum Fusobacteriota); *Escherichia* (phylum Pseudomonadota); and *Akkermansia* (phylum Verrucomicrobiota). In contrast, the GM of C-section-born infants consists of *Corynebacterium*, *Propiobacterium*, and *Slackia* (phylum Actinomycetota); *Staphylococcus*, *Streptococcus*, and *Veillonella* (phylum Bacillota); and *Enterobacter* and *Haemophilus* (phylum Pseudomonadota) [41,48,51,52,53,54]. Significant differences between the microbial content of meconium from vaginal and cesarean deliveries were reported by Martin et al. [36]. Meconium from C-section-born infants had lower bacterial levels than those from vaginally born neonates, with a marked decrease in species belonging to *Bifidobacterium*, *Bacteroides*, *Lactobacillus*, and *Enterococcus* and an increase in members of the genus *Clostridium* [36,48]. The different composition of the GM in C-section-born infants has been related to a higher risk of developing allergies, asthma, chronic immune disorders, type 1 diabetes, coeliac disease, obesity, and overweight later in life [55,56,57,58,59].

Breastfeeding provides a blend of nutrients that stimulate various important aspects, such as antimicrobial agents, bacterial growth, secreted IgA which fosters a regulatory immune system, and human milk oligosaccharides, recognized as prebiotics, that promote the growth and activity of beneficial microorganisms [43,60,61]. During the initial 3 months of life, the method of infant feeding by breastfeeding results in alterations in the GM composition, increasing the abundance of *Lactobacillus*, *Bifidobacterium*, *Enterococcus*, *Corynebacterium*, *Propiobacterium*, *Streptococcus*, and *Sneathia* genera and decreasing the abundance of *Bacteroides* and *Staphylococcus*. Infants fed by formula, however, exhibit a distinct GM composition, characterized by the abundance of bacterial genera *Atopobium* (phylum Actinomycetota); *Bacteroides* (phylum Bacteroidota); *Bilophila* (phylum Thermodesulfobacteriota); *Enterobacter*, *Escherichia*, and *Citrobacter* (phylum Pseudomonadota); and *Clostridium*, *Enterococcus*, *Lactobacillus*, and *Granulicatella* (phylum Bacillota) [36,54,62,63,64].

The administration of antimicrobials during the initial 3 years of life causes a reduction in the GM diversity and stability [65]. Antibiotics induce perturbations in the homeostasis of the GM with changes in the bacterial composition [66,67], such as a lower abundance of the genera *Bacteroides*, *Bifidobacterium*, *Lactobacillus*, *Staphylococcus*, and *Sediminibacterium* [36,53,68], and lead to an increase in some pathobionts, such as members of the family *Enterobacteriaceae* and the genus *Enterococcus* [68,69]. These perturbations can potentially negatively affect child health and increase their vulnerability to a number of microbial and neurocognitive disorders [70], such as ADHD, cognition disturbances, and depression [71]. Importantly, several central nervous system (CNS) processes, such as synaptogenesis, myelination, and synaptic pruning, that occur concurrently with GM maturation may be influenced by microbiome-associated metabolites [72].

During the weaning process, a diverse array of solid foods and novel nutrients are introduced, leading to an increase in microbial α-diversity and pH of the GM [41,73]. As a consequence, the dominant members of the infant microbiome shift from Actinomycetota and Pseudomonadota phyla to Bacillota and Bacteroidota phyla [30,74]. Additionally, a replacement of the dominant bacterial families occurs between 9 and 18 months of age, with an increase in the relative abundance of *Eubacteriaceae*, *Lachnospiraceae*, *Prevotellaceae*, *Rikenellaceae*, *Ruminococcaceae*, and *Sutterellaceae* [75]. Interestingly, at the genus level, a disparity was observed in the GM of infants who had ceased breastfeeding compared to those who were breastfed for an extended duration. In the former group, the most prevalent genera were *Akkermansia*, *Anaerostipes*, *Bacteroides*, *Bifidobacterium*, *Bilophila*, *Blautia*, *Clostridium*, *Faecalibacterium*, *Roseburia*, and *Ruminococcus*, while in the group of infants who were breastfed for a longer duration, the most abundant bacterial genera were *Collinsella*, *Lactobacillus*, *Megasphaera*, and *Veillonella* [41,53,73]. These microbial changes are associated with an increased protein intake (members of the *Lachnospiraceae*), an increased dietary fiber intake (members of the *Prevotellacea*), and an increase in mucin production (genus *Akkermansia*) [43]. The transition from a diet exclusively based on milk to the introduction of solid foods fosters the development of a mature microbiota that contains genes responsible for the degradation of complex carbohydrates and those responsible for vitamin production [30]. Afterward, the microbiota remains unstable until the infant reaches 2 to 3 years of age, a temporal moment at which the microbiota attains a composition analogous to that of adults [31,76].

The development and diversity of the GM appear to be closely related to geographic location and different lifestyles. A multicenter investigation performed in five European geographical contexts (Germany, Italy, Scotland, Spain, and Sweden) on more than 600 children approximately 4 weeks after the intake of the initial solid aliments compared to the same children before weaning (6 weeks of age), in which the influence of the type of food was excluded, showed that the feces of children born in northern countries contained a significantly higher prevalence of the genus *Bifidobacterium*, whereas the feces of children born in southern European countries were more diverse and showed a predominance of the members of the phylum Bacteroidota (*Lactobacillus* and *Bacteroides*) [74]. Another cohort study came to similar conclusions, but depending on the age [31], they showed that the GM typical of a US population was less diverse compared to the inhabitants of Venezuela and Malawi, but the differences were significant only in the group of children over 3 years of age and in adults. The authors also reported that GM composition was strongly associated with genes responsible for the metabolism of cobalamin (vitamin B_12_) and folic acid.

## 3. Influence of GM on the Brain Development

The influence of GM on the brain was suggested in 2011, when it was reported that germ-free (GF) mice showed an altered motor activity and a decreased anxiety-like behavior [77], and also by the manifestation of the anxiolytic and antidepressant properties of the probiotic *Lacticaseibacillus* (formerly *Lactobacillus*) *rhamnosus* strain JB-1 [78]. In addition, GF animals displayed disrupted spatial working and reference memory [79], impaired adult hippocampal neurogenesis [80], and reduced long-term potentiation in the CA1 region of the hippocampus [22]. Antibiotic treatment of mice changed GM and induced behavioral impairment by decreasing adult hippocampal neurogenesis and by altering synaptic transmission [81]. Interestingly, fecal microbiota transplantation from young mice donors reversed aging-associated cognitive impairment [24], and the reversal experiment induced cognitive behavioral deficits and a decrease in dendritic spines in the hippocampus and in the prefrontal cortex of young rats [82]. Several mechanisms through which alterations in the GM could impact brain plasticity have been suggested, such as the modulation of gene expression, the synthesis of neuroactive molecules, and the adjustment of microglial activity [83].

### 3.1. Gene Regulation by GM

The GM produces several metabolites that could reach distal tissues, such as the CNS, after release into the bloodstream, possessing the potential to shape the epigenetic mark [84]. Neuroepigenetics have a pivotal role in the CNS plasticity processes, and epigenetic changes occur on histone proteins and on DNA, including histone acetylation, DNA methylation, microRNA transcriptional silencing, and long noncoding RNA regulation [85].

Epigenetic regulation is governed by particular altering enzymes whose activity is controlled by microbial metabolites [86]. SCFAs may facilitate the acetylation of histone proteins, enabling the linking of transcription factors to DNA and thus promoting the transcription of genes [87]. The action of SCFAs is also important in the post-translational modification of histones called crotonylation [88,89]. In addition, SCFAs could promote plasticity in the visual cortex of adult mice, an outcome linked to alterations in microglial morphology [90], and affect gene expression on main cortical astrocytes [91].

Another epigenetic mark involved in neuronal plasticity that is associated with GM is the host DNA methylation. Modifications in DNA methylation occur during learning and memory consolidation, and its involvement in experience-dependent plasticity and regulation of synaptic function has been demonstrated [83,92]. The process of DNA methylation is influenced by the one-carbon metabolism pathway, which is regulated by the availability of cofactors required for DNA methyltransferases [93]; some of these cofactors are bacterial metabolites, such as cobalamin, folate, pyridoxine, and riboflavin [94,95]. However, although GM has been shown to induce DNA methylation changes in intestinal epithelial cells [96], there is no evidence yet suggesting a direct effect of microbiota on DNA methylation and transcription in the brain, nor is there a connection to changes in network plasticity [83].

MicroRNAs (miRNAs) are a group of small noncoding RNAs that function as post-transcriptional gene silencers. They attain this by interfering with target mRNAs and by inhibiting their role in various host systems, including the CNS [97]. Several studies have shown that the GM could modulate miRNA expression [98], which regulates various brain aspects and functions, such as dendritic morphology, spine density in hippocampal neurons, control of visual cortical plasticity (by interfering with spine remodeling), and regulation of cortical plasticity [99,100,101]. Preclinical studies have shown that the absence of the intestinal microbiota may be implicated in the changes of the expression of several miRNAs, particularly in the amygdala and in the prefrontal cortex, regions involved in anxiety and fear responses [102,103].

### 3.2. Regulation of Neuroactive Molecules by GM

The GM is capable of synthesizing several essential neurotransmitters for the CNS, such as gamma-aminobutyric acid (GABA), dopamine, histamine, and serotonin [104,105]. Some neurotransmitters, including dopamine and serotonin, are not able to cross the blood–brain barrier (BBB), and they need to be synthesized in the brain from their precursors (phenylalanine, tyrosine, tryptophan, and 5-hydroxytryptophan). The capacity of the GM to generate these precursor molecules, which can reach the brain, points to a potential contribution in determining cognitive function and behavior by influencing neuronal plasticity [104]. Serotonin, or 5-hidroxitriptamine (5-HT), regulates neuronal functions, including mood states, food desire, knowledge acquisition, memory and rest processes, and social behavior [106]. 5-HT is synthesized primarily from tryptophan in enterochromaffin cells of the gastrointestinal tract, although some evidence suggests that the GM may play a role in influencing brain serotonin production [105,107]. Moreover, serotonergic signaling plays a role in synaptic plasticity, influencing the regulation of long-term potentiation and depression, two critical aspects related to the consolidation of learning and memory processes [108]. In addition, Higa et al. [109] suggested that 5-HT plays a role in regulating synaptic plasticity in the prefrontal cortex during postnatal development. Yaghoubfar et al. [110] found that the administration of *Akkermansia muciniphila*-derived extracellular vesicles increased the concentration of 5-HT in the hippocampus and affected the expression of genes involved in 5-HT biosynthesis in the brain. Considering that extracellular vesicles released by bacteria have the capability to enter the bloodstream and cross the BBB, they may directly affect the regulation of other pathways in the CNS [111].

GABAergic transmission in the CNS is implicated in the modulation of both developmental and mature cortical plasticity [112,113]. Although several gut microbial groups produce GABA [114,115], there is no conclusive evidence for the influence of microbial GABA on brain plasticity [83]. However, bacterial-derived GABA may indirectly influence brain function by acting locally on the enteric nervous system or through the vagus nerve [116]. Interestingly, the administration of the probiotic *Lacticaseibacillus rhamnosus* affects the expression levels of GABA receptors in different subcortical brain areas, such as the amygdala, the hippocampus, and the locus coeruleus, and also in various cortical brain areas, such as the cingulate and the prelimbic cortex [78]. These changes were associated with a reduction in anxiety and in depressive-like behavior, with an improvement in memory formation during a fear-conditioning test, and with cognitive and emotional processes modulated by GABAergic transmission [117,118].

### 3.3. Modulation of Microglial Activity

Microglial cells constitute the only tissue-resident immune cells in the brain that are specialized in the response to a variety of immune-related stimuli and in the regulation of CNS plasticity, as well as learning and memory processes [119,120]. During brain development, microglia assist in both synapse removal and formation through physical interactions with synaptic structures or through diffusible factors [121,122,123,124]. In adulthood, microglia can regulate the synaptic plasticity resulting from experience [125] and several cognitive processes [123,126]. Erny et al. [19] presented the first evidence that the GM is pivotal for shaping the microglial phenotype, as in the absence of a GM, microglia displayed an immature signature and an altered morphology that can be reversed by microbial SCFAs administration. Furthermore, long-term absence of the maternal microbiome induced microglial changes during prenatal stages [127,128]. Acetate, an essential SCFA, drives microglial maturation and regulates the homeostatic metabolic state [129].

Several preclinical studies have reported that the interaction between the GM and microglia could be essential for the synaptic realignment in postnatal CNS development [130,131]. In addition, Bruckner et al. [132] found that impaired social behavior in animals was associated with a reduction in neurite complexity and with a less accurate innervation of forebrain neurons. This phenotype correlated with modifications in the quantity of microglial cells and in their expression of complement components, particularly C1q. Furthermore, microglia from germ-free mice or mice treated with antibiotics exhibited an immature transcriptomic signature, leading to inadequate dendritic spine remodeling and resulting in learning deficits [133]. Thus, microglia act as a link between the GM and brain plasticity; however, several factors, most notably diet, can alter the composition of the GM [33] and, consequently, impact the restructuring processes of neuronal networks by influencing microglial cells [134].

## 4. Neurodevelopmental Disorders (NDDs) in Children

Neurodevelopmental disorders (NDDs) are a set of specific conditions developed in early life that disrupt the brain development, altering cognitive, emotional, and motor functions [135,136]. Although the etiology of NDDs is not completely elucidated, evidence suggests that both genetic and environmental factors are involved in altering normal brain neurodevelopment, particularly neural plasticity [137,138]. The DSM-5 [139] includes as the most important NDDs the following: autism spectrum disorder (ASD), attention-deficit/hyperactivity disorder (ADHD), motor disorders (e.g., tic disorders such as Tourette syndrome), cerebral palsy, and fetal alcohol spectrum disorders (FASD). Some neurogenetic disorders, like the syndromes Rett, Down, Angelman, and Turner, have also been suggested to be included as NDDs [140].

Brain functions affected by NDDs when the individual develops and grows involve emotional response, self-control, learning capacity, intellectual performance, and social skills [139,140,141]. Quality of life (QoL) can be defined as “a person’s perception of his or her position in life” and includes different aspects such as an individual’s physical health, personal beliefs, psychological status, and social interactions [142]. The age of the patient is a key factor that may significantly influence the self-reported QoL [143,144,145]. In this respect, various studies revealed that the self-reported QoL of children with ADHD was similar to that of children with other NDDs or with serious diseases, such as cerebral palsy and cancer [145]. Additionally, other studies revealed that patients with ASD also presented very low self-reported QoL [144,146], but there were no differences found when compared to that of patients with ADHD [147].

### 4.1. Autism Spectrum Disorder (ASD)

Autism spectrum disorder (ASD) is characterized by deficits in social communication and restricted and/or repetitive behaviors, whose prevalence in children has been increasing in recent years [148]. Children with ASD exhibit additional dysfunctional behaviors, such as aggressivity, self-injury tendencies, and high levels of irritability [149]. The particular etiology of ASD is still unknown, although genetic, immune, and environmental aspects, as well as alterations in the GM, have been suggested as potential etiological factors [150,151,152,153]. Research has shown that GM changes have been reported in ASD children [154,155], resulting in neuroinflammation, alterations in neurotransmitters, increased oxidative stress, and intracellular acidification, which may lead to changes in the behavioral and functional development of children [156].

Finegold et al. [157] reported that the diversity of the GM in children with ASD was elevated, with an increase in the Bacteroidota phylum and a decrease in the Bacillota phylum, while Strati et al. [158] reported an increase in the Bacillota/Bacteroidota ratio in ASD subjects due to a decrease in the Bacteroidota abundance. The most abundant bacterial genera in ASD feces were *Lactobacillus*, *Desulfovibrio*, *Fusobacterium*, *Faecalibacterium*, *Collinsella*, *Corynebacterium*, *Dorea*, *Megamonas*, *Phyllobacterium*, *Coprobacter*, *Oscillospira*, *Ruminiclostridium*, *Barnesiella*, *Subdoligranulum*, *Klebsiella*, *Lactococcus*, *Lachnosbacterium*, *Lachnospira*, *Bacillus*, *Enterocloster*, and *Streptococcus*; furthermore, a decreased abundance was reported of the genera *Bifidobacterium*, *Flavonifractor*, *Dialister*, *Roseburia*, *Akkermansia*, *Alistipes*, *Eubacterium*, *Eisenbergiella*, *Blautia*, *Veillonella*, *Parasutterella*, *Haemophilus*, *Megasphaera*, *Turicibacter*, *Catenibacterium*, *Bradyrhizobium*, *Tyzzerella*, and *Intestinimonas* [152,157,158,159,160,161,162,163,164,165,166,167,168,169,170,171,172,173,174,175,176]. For the genera *Bacteroides*, *Prevotella*, *Sutterella*, *Ruminococcus*, *Bilophila*, *Parabacteroides*, *Escherichia/Shigella*, and *Clostridium*, controversial results were found [152,157,161,162,163,164,175,176]. In addition, several microbial taxa have been significantly associated with ASD core symptoms, such as *Bacteroides, Faecalibacterium*, and *Oscillospira* [160]; *Lachnoclostridium* and *Tyzzerella* [165]; and *Prevotella*, *Roseburia*, *Ruminococcus*, *Megasphaera*, and *Catenibacterium* [174] (Table 1).

Gut bacteria synthesize microbial metabolites and neurotransmitters that cross the gut barrier and the BBB, thereby affecting the CNS through mitochondrial function alteration and through the epigenetics of ASD-associated gene modulation [177]. However, the role of SCFAs in ASD is still controversial. In animal models, propionate induces gene expression and hippocampal histology and also neurobehavioral disturbances (repetitive actions and impaired social interaction) [178]. On the other hand, butyrate has a beneficial effect on social and repetitive behaviors in ASD-like mouse models [179]. In fact, butyrate may improve the BBB permeability and revert abnormalities in propionate-induced ASD [180]. In human studies, De Angelis et al. [163] found higher levels of propionate and acetate but lower levels of butyrate in children with ASD. In contrast, Liu et al. [167] found lower levels of fecal acetate and butyrate and higher levels of fecal valerate in ASD subjects. They also found a decreased abundance of butyrate-producing taxa (*Ruminococcaceae*, *Eubacterium*, *Lachnospiraceae*, and *Erysipelotrichaceae*) and an increased abundance of valerate-associated bacteria (members of Acidomycetota) in autistic individuals. In contrast, Wang et al. [181] reported that there was no significant association between the SCFAs levels and the risk of ASD.

Several studies have suggested a relationship between microbial neurotransmitters and the onset of symptoms related to ASD. Garcia-Gutierrez et al. [182] reported that ASD-associated symptoms, such as anxiety, cognitive deficits, and conduct disorder, may be related to the relative abundance of *Bifidobacterium* and *Bacteroides*, which are critical for GABA production [115]. The abundance of neurotoxin-producing *Clostridium difficile* and *C. histolyticum* was linked to ASD symptoms by Sivamaruthi et al. [183]. These toxins affect serotonin signaling, inducing ASD behaviors such as decreased socialization and response to pain, abnormal language, and self-injurious or repetitive behaviors [155,184]. Glutamate, which can be found in elevated levels in ASD children, acts as a neurotransmitter in the CNS and is implicated in the etiopathogenesis of several neurodevelopmental disorders [185]. Individuals with ASD have altered levels of p-cresol and p-cresyl sulfate that inhibit the enzyme dopamine-β-hydroxylase [186], potentially contributing to the behavioral and to the cognitive impairment symptoms caused by ASD [187].

### 4.2. Attention-Deficit/Hyperactivity Disorder (ADHD)

Attention-deficit/hyperactivity disorder (ADHD) is characterized by inappropriate and impairing signs of inattention, hyperactivity, and impulsivity [188]. This disorder is associated with remission or persistence into adulthood depending on various childhood risk factors [189]. The exact etiology of ADHD remains unknown, although it has been suggested that its development and symptoms may be related to the modulation of the GM exerted by diets or by early GM composition [190,191,192].

Several studies have been conducted on the GM of ADHD individuals, obtaining conflicting results [193] (Table 2). The bacterial richness and evenness (α diversity) and the community structure (β diversity) decrease in ADHD individuals. Additionally, an increase in members of the families *Enterococcaceae*, *Lachnospiraceae*, *Moraxellaceae*, *Neisseriaceae*, *Odoribacteriaceae*, *Peptococcaceae*, *Peptostreptococcaceae*, *Selenomonadaceae*, *Veillonellaceae*, and *Xanthomonadaceae* has been reported, while members of the families *Alcaligenaceae*, *Catabacteriaceae*, *Gracilibacteriaceae*, *Muribaculaceae*, *Prevotellaceae*, *Porphyromonadaceae*, and *Ruminococcaceae* showed decreased abundance [194,195,196,197,198]. The predominant genera in the GM of ADHD subjects were *Agathobacter*, *Akkermansia*, *Anaerostipes*, *Bifidobacterium*, *Blautia*, *Collinsella*, *Dorea*, *Eggerthella*, *Escherichia/Shigella*, *Fusobacterium*, *Intestinibacter*, *Megamonas*, *Neisseria*, *Odoribacter*, *Phascolarctobacterium*, and *Roseburia*, while the genera *Anaerotaenia*, *Coprococcus*, *Faecalibacterium*, *Gracilibacter*, *Lachnosclostridium*, and *Ruminococcus* were reduced compared to the controls [190,192,194,195,196,197,198,199,200]. Conflicting results have been reported for the genera *Bacteroides*, *Dialister*, *Enterococcus*, *Parabacteroides*, *Prevotella*, and *Sutterella* in different studies [192,194,196,197].

Various studies have reported relationships between microbial taxa, ADHD symptoms, and altered metabolic pathways. Aarts et al. [199] found a correlation between *Bifidobacterium* levels and the abundance of phenylalanine pathway enzymes in patients with ADHD. Swann et al. [206] found that the fecal profile of individuals with ADHD presented metabolic perturbations due to gene variants previously associated with behavioral symptoms in this disorder. Negative correlations have been found between the hyperactivity and the *Faecalibacterium* abundance [195] and between the *Coprococcus* abundance and the inattention scores [200]. In addition, *Bacteroides* was related to the levels of hyperactivity and of impulsivity in ADHD children [192,195]. Moreover, differences in the neurotransmitter metabolic pathways have been also associated with ADHD patients [197].

### 4.3. Tic Disorders (TDs) and Tourette Syndrome (TS)

Tic disorders (TDs) are NDDs with childhood onset, characterized by sudden, repetitive, and nonrhythmic movements that can persist into adulthood [207]. Tourette syndrome (TS) is the most severe form of TD. Specifically, TS can be defined as a chronic NDD that typically begins in childhood and is characterized by motor and phonic tics, which can significantly reduce the quality of life of affected individuals [208,209]. Although most patients generally can recover on their own before reaching maturity, approximately one-third of them will continue to have symptoms into adulthood. TS with acute symptoms can present unmanageable behavior and obscene speech (coprolalia), which highly interfere with the quality of life and with the social dynamics of individuals suffering from this syndrome [210]. Although the pathophysiology of TS remains unknown, several authors have suggested various interactions with genetic, neurobiochemical, immunological, microbial, and environmental factors [211,212,213,214,215].

The possible relationship between the TS symptom severity and the alteration of GM in TS patients has recently been reviewed [216,217] (Table 2). No consensus was reported in α- and β-diversity among the studies reviewed. One study found a decrease in members of the family *Prevotellaceae* and of the genus *Prevotella*, while the genus *Ruminococcus* was increased in TS patients. At the species level, a decrease in the abundance of *Clostridium bartlettii*, *Prevotella copri*, and *Subdoligranulum variabile* was also reported [201]. In addition, these authors found a negative association between the abundance of the genus *Prevotella* and the severity of the TS symptoms. In another study, the species *Allisonella histaminiformans*, *Bacteroides coprocola*, *Catenibacterium mitsuokai*, *Dialister succinatiphilus*, *Holdemanella biformis*, and *Roseburia faecis* showed lower abundance in the GM of the TS individuals, while only *Bacteroides vulgates* increased [202]. More recently, Xi et al. [203] found significantly higher abundance of *Bacteroides plebeius* and *Ruminococcus lactaris* and lower abundance of *Prevotella stercorea* and *Streptococcus lutetiensis* in TS children; furthermore, they found that drug treatment with a dopamine receptor antagonist results in the restoration of the GM dysbiosis. In addition, a significantly higher abundance of *B. plebeius* and *R. lactaris* in treatment-naïve children was found, suggesting that these bacterial genera and their metabolites may be involved in oxidative stress and in the production of inflammatory factors. GABA degradation was also significantly increased in TS children, and *Klebsiella pneumoniae*, a GABA-degrading bacterial species, showed a positive correlation with the worsening of TS symptoms, while *Eubacterium* spp., *Bifidobacterium* spp., and *Akkermansia muciniphila*, which are thought to be associated with the production of GABA, exhibited a more pronounced negative correlation with the YGTSS score. On the contrary, several *Bacteroides* species (*B. eggerthii*, *B. dorei*, and *B. thetaiotaomicron*) showed positive correlations with YGTSS scores.

### 4.4. Cerebral Palsy and Epilepsy

Cerebral palsy (CP) is the most frequently childhood disability affecting motor function [218]. The symptoms of CP are heterogeneous but usually consist of permanent disturbances in the development of movement and posture, resulting in activity limitations [219]. Approximately 40% of CP patients often have other concurrent cerebral neurological disorders such as epilepsy (CPE) [220], with its incidence in CP patients being five times higher than in healthy children [221]. Neonatal convulsions, low birth weight, intracranial hemorrhage, gray and white matter brain lesions, and malformations of the brain structure are the major risk factors for epilepsy in CP children [222].

Children with CPE showed higher microbial diversity and different bacterial profiles in their GM compared to healthy children [204]. However, it is difficult to attribute these changes to CPE or to the distinct lifestyle and dietary factors associated with these interactive neurological conditions [223]. These authors found an alteration in the GM of CPE patients [204,223]. At the phylum level, the relative abundance of members of Actinomycetota is significantly increased, while the relative abundance of Bacteroidota is significantly decreased in these patients. At the genus level, the relative abundance of the beneficial bacteria *Bifidobacterium* and the opportunistic pathogenic bacteria *Enterococcus*, *Parabacteroides*, and *Streptococcus* significantly increases, but the relative abundance of butyric acid-producing bacteria such as *Bacteroides*, *Faecalibacterium*, *Ruminococcus*, and *Roseburia* significantly decreases (Table 2). These GM changes induce a chronic inflammation in the intestinal tract and gastrointestinal disorders such as functional constipation, as well as a reduction in the biosynthesis of secondary microbial metabolites.

Peng et al. [205], comparing a group of CPE patients with controls, found a significantly lower abundance of *Bacteroides fragilis* and *Dialister invisus* but a higher abundance of *Phascolarctobacterium faecium*, *Eubacterium limosum*, and *Veillonella* sp. (Table 2). In terms of microbiome functional pathways, CPE subjects showed a decrease in pathways for degradation of serine, quinolinic acid, glutamate, and glycerol and dissimilatory reduction of sulfate and nitrate but an increase in pathways related to ethanol production. Interestingly, both glutamate and serine are agonists of the *N*-methyl-D-aspartate receptors, and the anomalous expression or function of these receptors may be at the root of the pathophysiology of seizure disorders and epilepsy [224,225].

### 4.5. Fetal Alcohol Spectrum Disorders (FASD)

Fetal alcohol spectrum disorders (FASD) refers to a wide set of impairments that occur in children as a result of their mothers’ alcohol consumption during pregnancy, the most severe of which is fetal alcohol syndrome (FAS) [226]. FASD are associated with abnormalities in brain structure and development, white matter microstructure, and functional connectivity that alter developmental trajectories and result in deficits in cognition, executive function, memory, vision, hearing, motor skills, behavior, and social adjustment [227].

Alcohol easily passes through the placenta and can interfere with fetal development. Damage provoked by prenatal alcohol exposure (PAE), which can cause FASD, depends on the dose, pattern, timing, and duration of the exposure; fetal and maternal genetics; maternal nutrition and substance use; and epigenetic responses [228]. PAE can affect brain development [229], causing microcephaly, hydrocephalus, corpus callosum defects, prenatal ischemic lesions, small subarachnoid heterotopias, holoprosencephaly, and lissencephaly [230].

PAE may cause permanent changes in the GM, as demonstrated in preclinical studies, and an increase in the α- and β-diversity. Virdee et al. [231] reported that PAE altered the biochemical profile within the maternal–fetal dyad, and this metabolite profile is derived from the GM due to the alteration caused by alcohol. In addition, Bodnar et al. [232] found that PAE rats had a greater abundance of bacterial species, with the genera *Bacteroides*, *Roseburia*, and *Proteus* being the most abundant. In addition, significant sex-specific differences were also observed for several bacterial genera; in males, *Bacteroides*, *Bifidobacterium*, *Akkermansia*, and *Ruminiclostridium* and in females, *Bacteroides*, *Roseburia*, *Faecalitalea*, and *Proteus* were found to be significantly altered by PAE. However, more research is needed that considers the fetal microbiota in the development of new interventions for FASD.

## 5. Genetic NDDs

### 5.1. Rett Syndrome (RTT)

Rett syndrome (RTT) is an NDD that is more common in girls and that is attributed to spontaneous and noninherited mutations, involving the methyl-CpG binding protein 2 (*MeCP2*) gene located on the X chromosome [233]. RTT is mainly characterized by loss of acquired language and motor skills, repetitive hand movements, respiratory irregularities, and seizures, symptoms that begin to manifest in early childhood and progress through several later stages [234].

Female RTT patients are characterized by altered gastrointestinal homeostasis and hypomotility, resulting in gastrointestinal disturbances [235]. The occurrence of an intestinal subinflammatory status and the subsequent change in the relative abundance of GM components in RTT patients have been reported [236] (Table 3). The former authors reported the existence of altered bacterial and fungal microbiota in RTT, dominated by *Actinomyces*, *Anaerostipes*, *Bifidobacterium*, *Clostridium*, *Eggerthella*, *Enterococcus*, *Escherichia/Shigella*, *Lactobacillus*, members of *Erysipelotrichaceae*, and the fungal genus *Candida*. This GM dysbiosis also induced an alteration in the SCFA metabolite profile (enriched in propionate, isobutyrate, and isovalerate-2-methylbutyrate), which may be related to the constipation symptoms and gastrointestinal pathophysiology present in RTT patients.

GM dysbiosis, its metabolic products, and diet seem to be the main factors involved in the pathophysiology and disease severity in RTT patients [237]. These authors found that the GM of RTT patients had a reduction in α diversity, and the microbiota was dominated by members of the phyla Bacteroidota and Bacillota and of the families *Bacteroidaceae*, *Ruminococcaceae*, *Erysipelotrichaceae*, and *Lachnospiraceae.* At the genus level, *Bacteroides*, *Clostridium*, and *Sutterella* were the most abundant, while *Faecalibacterium*, *Prevotella*, and *Roseburia* were slightly depleted. Correlation analysis between diet and microbiota showed that *Bacteroides* and *Clostridium* were positively associated with total protein and animal protein intake, while fiber intake was positively associated with members of the family *Christensenellaceae*.

Recently, Thapa et al. [238] investigated the relationship between gut bacterial dysbiosis and gastrointestinal dysfunction in different RTT phenotypes. The predominant phyla obtained were Bacillota and Bacteriodota, and the major families reported were *Bacteroidaceae*, *Lachnospiraceae*, *Ruminococcaceae*, *Bifidobacteriaceae*, *Verrucomicrobiaceae*, *Porphyromonadaceae*, and *Prevotellaceae*. Although differences were found between the RTT and control groups in the genera *Bacteroides*, *Parabacteroides*, and *Clostridium* (increases) and *Prevotella* and *Faecalibacterium* (decreases), these differences were not significant. The changes in the GM of RTT were correlated with the clinical phenotype of the disease. Thus, the age of the RTT cohort tended to correlate inversely with the following parameters: α-diversity of the GM and height and weight of the RTT individuals. On the other hand, phenotypic markers such as race, body mass index, mutation severity, several clinical symptoms (anxiety, seizures, bruxism, hyperventilation, abdominal distension), bowel movement frequency, stool consistency, and medication use had no effect on the GM β-diversity in the RTT cohort. The microbial community composition in the RTT cohort was significantly different depending on the pubertal status and the clinical severity score. In addition, concentrations of selected end products of the gut bacterial metabolome were lower in the RTT cohort. Therefore, a causal relationship between gut bacterial dysbiosis and gastrointestinal dysfunction could not be established; nevertheless, the pathophysiology of gastrointestinal problems in RTT could be related to the gut metabolome.

### 5.2. Down Syndrome (DS)

Down syndrome (DS) consists of a set of clinical features resulting from a trisomy of chromosome 21 and represents a genetically complex condition that is compatible with human postnatal survival, for which it is the most common autosomal aneuploidy with survival [242]. The overall increase in life expectancy in DS is accompanied by a parallel increase in the risk of age-related diseases such as Alzheimer’s and Parkinson’s diseases and increased rates of infection, hypertension, and obesity [243,244,245]. The acceleration of biological aging in DS mainly involves an increase in the oxidative stress and a premature immunosenescence in the immune system [246].

Previously, human aging and immunosenescence have been linked to a deterioration of the mutualistic relationship with the GM [247]. For this reason, several studies have been performed to determine the influence of DS on the GM dysbiosis (Table 3). Biagi et al. [239] found specific signatures in the GM of DS individuals, such as an increase in β-diversity and in the abundance of the phyla Bacillota (families *Ruminococcaceae*, *Lachnospiraceae*, and *Clostridiaceae*), Actinomycetota (family *Bifidobacteriaceae*), and Bacteriodota (family *Bacteroidaceae*), with the most abundant genera being *Parasporobacterium* and *Sutterella*. In contrast, it a relative decrease was found in the α-diversity and in the abundance of members of the family *Veillonellaceae*. Only the abundance of *Sutterella* was significantly correlated with the Aberrant Behavior Checklist total scores, which may suggest a possible role for this genus in the behavioral features related to DS.

Ren et al. [240] conducted a case-control study to investigate the GM composition in Chinese DS children and the relationships between changes in GM composition and DS cognitive function scores. Both the shape and richness of the GM differed between DS children and healthy controls. The richness of the *Acidaminococcaceae* family was decreased in DS children. At the genus level, the most notable differences were the lower relative abundance of *Collinsella*, *Coprobacillus*, *Klebsiella*, *Megamonas*, *Prevotella*, *Ruminiclostridium*, *Slackia*, and *Tyzzerella* in the DS group, while the genera *Anaerostipes*, *Faecalibacterium*, *Phascolarctobacterium*, and *Turicibacter* were higher in the DS patients than in the healthy controls. In addition, the genera *Blautia* and *Citrobacter* were negatively associated with cognitive scores in DS.

Interestingly, based on the previously reported association of the genus *Blautia* with cognitive function in these children [248], Hou et al. [243] performed *Blautia*-specific amplicon sequencing to identify the specific *Blautia* species in 15 children with DS and 15 healthy children. These authors found differences in the abundance of the *Blautia* species between the intervention group, with *B. massiliensis* and *B. argi* decreasing in DS children and *B. faecis* increasing. In addition, *B. argi* was positively associated with DS cognitive scores, and *B. faecis* was negatively related to cognitive function, suggesting a role in DS cognitive impairment. 

### 5.3. Angelman and Turner Syndromes

Angelman syndrome (AS) is a rare NDD resulting from a maternally inherited deletion of ubiquitin ligase E3A (*UBE3A* gene) gene expression in brain neurons, and it is characterized by microcephaly, severe intellectual disability, speech and language impairment, epilepsy, ataxia, tongue protrusion, laughing fits, abnormal sleep patterns, and hyperactivity [248,249].

In addition to neurodevelopmental effects, gastrointestinal disturbances are also frequently reported in AS patients [250]. To date, only preclinical studies have been performed on the alteration of the GM in AS. Thus, Beitnere et al. [251] identified changes in the bacterial abundance of the GM in three animal models of AS (mouse, rat, and pig). At the phylum level, the GM of AS animal models shows an increased abundance of Bacteroidota and Actinomycetota and a decreased abundance of Bacillota compared to controls, with a reduction in the Bacteroidota/Bacillota ratio. At the genus level, AS animal models show a decreased abundance of *Lactobacillus* (mice), *Dubosiella* (mice), *Streptococcus* (pigs), *Akkermansia* (rats), *Eubacterium* (rats), *Clostridium* (pigs), and *Fibrobacter* (pigs) compared to controls but increased levels of *Bacteroides* (mice and rats), *Blautia* (rats and pigs), *Faecalibacterium* (mice and pigs), *Lachnospiraceae incertae sedis* (mice), *Helicobacter* (mice), *Turicibacter* (mice), *Marvinbryantia* (mice), *Roseburia* (rats), *Ruminococcus* (rats), *Nocardia* (rats), *Subdoligranulum* (pigs), *Treponema* (pigs), and *Butyricicoccus* (pigs). Controversial results were obtained for the genera *Monoglobus* and *Tepidibacter*. Interestingly, certain bacterial groups were significantly increased in all animal models, including *Lachnospiraceae incertae sedis*, *Desulfovibrio*, and *Odoribacter*, which have been correlated with neuropsychiatric disorders.

Turner syndrome (TUS) is the most common sex chromosome abnormality in females [252], and it is associated with either complete or partial loss of an X chromosome, often with a mosaic karyotype [253]. Clinical features of TUS involve multiple organ systems and include short stature; dysmorphic facial features; delayed puberty and gonadal failure; cardiac, renal, and audiologic abnormalities; and a high prevalence of endocrine and autoimmune disorders [254]. Although some studies have shown that girls with TUS have periodontal problems [255,256], no study to date has linked this syndrome to the GM. Ünsal et al. [241], using microarray analysis, found that *Actinomyces viscosus*, *Aggregatibacter actinomycetemcomitans*, *Eikenella corrodens*, *Fusobacterium nucleatum*, and *Prevotella intermedia* were the dominant species in the saliva of the TUS group.

## 6. Future Perspectives and Conclusions

The pathophysiology of NDDs is not completely understood at present. However, there is a general consensus supporting the hypothesis that the development of human NDDs is strongly linked to the quality of the prenatal lifestyle [257]. The development of the brain during the fetal stage and during the first years of life is highly vulnerable to external factors. Hence, distressing events in this period may alter brain structure and function, increasing susceptibility to NDDs [258]. Brain–GM interactions are developed in the early years of childhood and may be modulated by diet, drugs, and stress throughout life [259]. There are several ways in which the GM can affect human behavior, mainly through the nervous system, immune system, tryptophan metabolism, and HPA axis [8]. The effects of the GM are mediated through the synthesis of microbial metabolites, neurotransmitters, and certain metabolic pathways that may play a role in NDDs. However, the exact role of the GM in modulating the symptoms of NDDs remains a highly controversial topic. Therefore, studies should be conducted to gain a better understanding of involvement of the GM in the development and onset of NDDs. As the symptomatology of NDDs is quite broad, future research should be based on a more holistic approach, such as the study of the role of the GM and its composition on the pathophysiology of NDDs or even the therapeutic interventions in NDDs [141]. Nevertheless, establishing a definitive cause–effect relationship between the altered GM and the disease can be quite demanding, since it is not easy to determine whether the GM changes are casual or consequential responses to it.

For this reason, a wider comprehension of the role of the GM on human behavior is needed to elucidate its implications within the clinical and social sciences. The mechanisms of action and the extent to which bacteria and their metabolites may influence brain functions are poorly known; however, this deficiency could be resolved with new advances in metabolomics technology. In this sense, new high-throughput sequencing technologies will facilitate a deeper knowledge of the GM composition and of its association with mental disorders [260]. In particular, new and noninvasive sampling devices will allow the collection of luminal contents throughout the intestinal tract, avoiding the limitations of stool sampling [261].

It should be highlighted that NDDs have profound implications for society, affecting not only those diagnosed individuals but also their families and communities. These disorders can generate important challenges in different areas, such as satisfactory social interaction, effective communication, and adaptive behavior, which significantly affect the quality of life of affected children. In this respect, the symptoms and outcomes of individuals with NDDs are strongly influenced by contextual and social factors, including family dynamics, educational environments, cultural norms, community awareness, and socioeconomic status. Understanding these influences is crucial, as enriched and supportive environments can mitigate symptoms and improve adaptive functioning. Therefore, it is imperative to foster collaboration between researchers from clinical and social fields. Such interdisciplinary cooperation may lead to a more holistic understanding of the multifaceted nature of NDDs and their correlates, which will ultimately promote the progress on comprehensive intervention initiatives, providing a greater community inclusion of these groups, a higher visibilization of their necessities, and an enhancement of their well-being.

In conclusion, the worldwide prevalence of NDDs combined with the lack of optimal pharmacological treatments emphasizes the need for alternative strategies, such as the implementation of new therapeutic interventions of NDDs targeting the GM. In this sense, the use of psychobiotics, fecal microbiota transplantation (FMT), or personalized diets has opened new perspectives to treat NDDs and reduce the symptoms of these disorders. In addition, large randomized trials are required to clarify the long-term efficacy and the potential side effects of these therapeutic tools. Such therapeutic interventions consist of the modulation of the human GM, which could offer relief of clinical symptoms and a promising enhancement in the quality of life for individuals with NDDs.

## Figures and Tables

**Table 1:** Changes in bacterial GM composition of individuals with ASD.

Studies	Country/Year	Participants	Increased Genera	Decreased Genera
Parracho et al. [170]	UK/2005	58 ASD	*Clostridium*	
Finegold et al. [157]	USA/2010	33 ASD	*Clostridium* *Desulfovibrio*	
Adams et al. [159]	USA/2011	58 ASD	*Lactobacillus*	*Bifidobacterium*
Finegold et al. [152]	USA/2012	13 ASD	*Clostridium*	
De Angelis et al. [163]	Italy/2015	10 ASD	*Clostridium*	*Bifidobacterium*
Pärty et al. [171]	Finland/2015	6 ASD		*Bifidobacterium*
Tomova et al. [172]	Slovakia/2015	10 ASD	*Lactobacillus* *Desulfovibrio*	
Strati et al. [158]	Italy/2017	40 ASD	*Collinsella* *Corynebacterium* *Dorea* *Lactobacillus*	*Alistipes* *Bilophila* *Dialister* *Parabacteroides* *Veillonella*
Liu et al. [167]	China/2019	30 ASD	*Barnesiella* *Coprobacter* *Fusobacterium*	*Eubacterium*
Ma et al. [168]	China/2019	45 ASD	*Enterocloster*	*Fravonifractor* *Tyzzerella*
Niu et al. [169]	China/2019	114 ASD	*Lachnospira*	*Bacteroides* *Bifidobacterium* *Blautia* *Roseburia* *Ruminococcus*
Zhai et al. [175]	China/2019	78 ASD	*Bacillus* *Bacteroides* *Bilophila* *Lachnosbacterium* *Lachnospira* *Lactococcus* *Oscillospìra* *Parabacteroides* *Sutterella*	
Dan et al. [162]	China/2020	143 ASD		*Bacteroides* *Prevotella* *Sutterella*
Wu et al. [174]	China/2020	169 ASD		*Catenibacterium* *Megasphaera* *Prevotella* *Roseburia* *Ruminococcus*
Zou et al. [176]	China/2020	48 ASD	*Bacteroides* *Megamonas* *Prevotella*	*Akkermansia* *Clostridium* *Dialister* *Eisenbergiella* *Escherichia/Shigella* *Fravonifractor* *Haemophilus*
Chen et al. [160]	China/2021	138 ASD	*Bacteroides* *Faecalibacterium*	*Prevotella*
Ding et al. [164]	China/2021	25 ASD	*Faecalibacterium* *Subdoligranulum*	*Bifidobacterium*
Huang et al. [165]	China, USA/2021	39 ASD	*Escherichia/Shigella* *Phyllobacterium*	
Chen et al. [161]	Taiwan/2022	82 ASD	*Bacteroides* *Fusobacterium* *Ruminococcus*	*Clostridium* *Intestinimonas* *Parasutterella* *Turicibacter*
Wang et al. [173]	China/2023	42 ASD	*Ruminiclostridium* *Ruminococcus* *Streptococcus*	*Bradyrhizobium* *Fravonifractor*
Zuffa et al. [166]	Sweden/2023	16 ASD	*Clostridium* *Klebsiella*	*Bifidobacterium*

**Table 2:** Changes in bacterial GM composition of individuals with several NDDs.

Studies	Country/Year	Participants	Increased Genera	Decreased Genera
Aarts et al. [199]	The Netherlands /2017	19 ADHD	*Bifidobacterium* *Eggerthella*	
Jiang et al. [194]	China/2018	51 ADHD		*Dialister* *Faecalibacterium* *Lachnosclostridium* *Sutterella*
Prehn-Kristensen et al. [195]	Germany/2018	14 ADHD	*Bacteroides* *Neisseria*	*Parabacteroides* *Prevotella*
Szopinska-Tokov et al. [200]	The Netherlands /2020	42 ADHD	*Intestinibacter*	*Coprococcus* *Prevotella*
Wan et al. [197]	China/2020	17 ADHD	*Bacteroides* *Enterococcus* *Odoribacter*	*Faecalibacterium* *Ruminococcus*
Wang et al. [192]	Taiwan/2020	30 ADHD	*Bacteroides* spp. *Escherichia/Shigella* *Fusobacterium* *Parabacteroides* *Phascolarctobacterium* *Prevotella* *Sutterella*	*Bacteroides* spp.
Richarte et al. [196]	Spain/2021	100 ADHD	*Dialister* *Megamonas*	*Anaerotaenia* *Gracilibacter*
Wang et al. [198]	Taiwan/2022	41 ADHD	*Agathobacter* *Anaerostipes* *Roseburia*	
Cassidy-Bushrow et al. [190]	USA/2023	59 ADHD	*Akkermansia* *Blautia* *Collinsella* *Dorea*	*Enterococcus* *Ruminococcus*
Lee and Wong [201]	Taiwan/2018	14 TS	*Ruminococcus*	*Clostridium* *Prevotella* *Subdoligranulum*
Zhao et al. [202]	China/2020	5 TS	*Bacteroides*	*Allisonella* *Catenibacterium* *Dialister* *Holdemanella* *Roseburia*
Xi et al. [203]	China/2021	49 TD	*Bacteroides* *Ruminococcus*	*Prevotella* *Streptococcus*
Huang et al. [204]	China/2019	25 CPE	*Akkermansia* *Bifidobacterium* *Clostridium* *Enterococcus* *Prevotella* *Rothia* *Streptococcus* *Veillonella*	*Anaerostipes* *Bacteroides* *Blautia* *Faecalibacterium* *Parasutterella* *Roseburia* *Ruminococcus*
Peng et al. [205]	Hong Kong (China)/2023	13 CPE	*Eubacterium* *Phascolarctobacterium* *Veillonella*	*Bacteroides* *Dialister*

ADHD: Attention-Deficit/Hyperactivity Disorder; TS: Tourette syndrome; TD: Tic Disorder; CPE: Cerebral Palsy and Epilepsy.

**Table 3:** Changes in bacterial GM composition of individuals with various genetic NDDs.

Studies	Country/Year	Participants	Increased Genera	Decreased Genera
Strati et al. [236]	Italy/2016	50 RTT	*Actinomyces* *Anaerostipes* *Bifidobacterium* *Clostridium* *Eggerthella* *Enterococcus* *Escherichia/Shigella* *Lactobacillus*	
Borghi et al. [237]	Italy/2018	8 RTT	*Bacteroides* *Clostridium* *Sutterella*	*Faecalibacterium*
Thapa et al. [238]	USA/2021	44 RTT	*Bacteroides* *Clostridium* *Parabacteroides*	*Faecalibacterium* *Prevotella*
Biagi et al. [239]	Italy/2014	17 DS	*Parasporobacterium* *Sutterella*	
Ren et al. [240]	China/2022	15 DS	*Anaerostipes* *Blautia* *Faecalibacterium* *Phascolarctobacterium* *Turicibacter*	*Collinsella* *Coprobacillus* *Klebsiella* *Megamonas* *Prevotella* *Ruminiclostridium* *Slackia* *Tyzzerrella*
Ünsal et al. [241]	Turkey/2019	20 TUS *	*Actinomyces* *Aggregatibacter* *Eikenella* *Prevotella*	

RTT: Rett syndrome; DS: Down syndrome; TUS: Turner syndrome. * Oral microbiome.

## Data Availability

Not applicable.
